# Forensic Analysis of Textile Synthetic Fibers Using a FT-IR Spectroscopy Approach

**DOI:** 10.3390/molecules27134281

**Published:** 2022-07-03

**Authors:** Abdulrahman Aljannahi, Roudha Abdulla Alblooshi, Rashed Humaid Alremeithi, Ioannis Karamitsos, Noora Abdulkarim Ahli, Asma Mohammed Askar, Ikhlass Mohammed Albastaki, Mohamed Mahmood Ahli, Sanjay Modak

**Affiliations:** 1 Dubai Police General Headquarters, Dubai 1492, United Arab Emirates; abdulrahmanaljanaahi@gmail.com (A.A.); ra.alblooshi@dubaipolice.gov.ae (R.A.A.); r.huzaim@dubaipolice.gov.ae (R.H.A.); n.ahli@dubaipolice.gov.ae (N.A.A.); frn.aalmardaei@dubaipolice.gov.ae (A.M.A.); ibastaki@dubaipolice.gov.ae (I.M.A.); m.mmahli@dubaipolice.gov.ae (M.M.A.); 2 Research and Graduate Department, Rochester Institute of Technology, Dubai 1492, United Arab Emirates; ssmcada@rit.edu

**Keywords:** textile fibers, spectroscopy, FT-IR, forensic, PCA, SIMCA

## Abstract

Synthetic fibers are one of the most valuable trace lines of evidence that can be found in crime scenes. When textile fibers are analyzed properly, they can help in finding a linkage between suspect, victim, and the scene of the crime. Various analytical techniques are used in the examination of samples to determine relationships between different fabric fragments. In this exploratory study, multivariate statistical methods were investigated in combination with machine learning classification models as a method for classifying 138 synthetic textile fibers using Fourier transform infrared spectroscopy, FT-IR. The data were first subjected to preprocessing techniques including the Savitzky–Golay first derivative method and Standard Normal Variate (SNV) method to smooth the spectra and minimize the scattering effects. Principal Component Analysis (PCA) was built to observe unique patterns and to cluster the samples. The classification model in this study, Soft Independent Modeling by Class Analogy (SIMCA), showed correct classification and separation distances between the analyzed synthetic fiber types. At a significance level of 5%, 97.1% of test samples were correctly classified.

## 1. Introduction

During a forensic investigation, any micro-level evidence that can be transferred between persons, locations, and objects is generally called trace evidence. According to the Locard principle, the purpose of using trace evidence is to find a link between suspects, victims, and crime scenes. Trace evidence can include a wide range of materials, such as: fibers, paint, glass, gunshot residue, soil, and others [1,2]. Textile fibers are one of the most crucial pieces of evidence that can be easily found at crime scenes. They have been classified into natural or man-made fiber, based on physical and chemical properties. Fiber can be identified by performing a proper comparison using certain analytical techniques [1].

Fiber examination techniques can be divided into destructive and non-destructive techniques, according to the damage caused after the analysis. One of the most common sophisticated techniques that is considered as destructive is the coupling of chromatographic techniques and mass spectrometer. According to the nature of the mobile phase, chromatographic techniques can be classified as Liquid chromatography (LC) and Gas chromatography (GC). LC–MS is commonly used to separate and identify dye components in trace fibers. Can Hu et al. developed a sensitive LC–MS/MS method for the analysis of fiber dyes in different dye classes using a Multi Reaction Monitor (MRM) that is capable of analyzing single fibers with length of few millimeters [3]. Although some fibers share the same dye color, it has been proved that it is possible to distinguish between them due to their differences in dye chemistry. A case study was carried out by a group of researchers from the Netherlands Forensic Institute to identify fiber dyes in seven different cases using LC–MS. Most fiber samples had been identified even when they had the same color, highlighting the importance of fiber examination as forensic evidence [4]. On the other hand, the composition of fiber itself can be identified by using thermal energy to break down the fiber chemical bonds, leading to molecular fragments in a technique called pyrolysis–gas chromatography/mass spectrometry (Py–GC/MS). Back in 2006, Valerio Causin et al. were able to distinguish between colorless acrylic fibers using Py–GC/MS [5]. Recently, Se Jeong Lim et al. proposed a validated Py–GC/MS method for the analysis of microfibers (MFs) such as: nylon-6, polyethylene terephthalate, and polyacrylonitrile, emitted from textiles during the laundering process. They were able to identify and quantify different types of filtered MFs in wastewater successfully [6].

On the other hand, there are various non-destructive analytical techniques that can be used to examine fiber samples, such as: microscopy, spectroscopy, and ultraviolet−visible microspectrophotometry (UV−vis MSP). Microscopy has a wide range of applications in forensic science because of its ability to identify, resolve, and photograph small pieces of fibers without requiring any structural changes. Fiber surface characteristics, diameters, and optical properties such as refractive index can be obtained by polarized light microscopy. Furthermore, an electron microscope can be used, as well, since it has the advantages of a wide range of magnification and performing elemental analysis when it is coupled with an energy dispersive X-ray spectrometer [1]. In Western Australia, the MSP textile fiber database has been created for forensic cases that spanned 24 years by Rees Powell et al., specifically for the interpretation of data in connection with the investigation. Interestingly, they found that textile fiber was critical evidence in three offence cases, where, in one of the homicide cases, the recovered fiber played an essential role in the investigation [7].

Spectroscopic techniques can be used for fiber analysis, such as: Raman spectroscopy, Fourier transform infrared spectroscopy (FT-IR), and X-ray fluorescence spectroscopy (XRF). J Zieba et al. evaluated the application of the new combined µ-Raman and µ-XRF spectrometer in analysis of fibers and other trace evidence, where the Raman spectra of fibers mainly provide information about the polymer matrix and some pigments [8]. A recent study proposed the use of Raman imaging with different methods of multivariate chemometric analysis, concluding its ability to discriminate textile fibers [9]. In fact, there are some limitations where textile dyes represent an obstacle, since most Raman bands came from dye but not from the fiber itself [10,11,12,13]. In contrary, FT-IR is more useful for distinguishing between fibers belonging to the same generic class and subclass. Furthermore, this method is found to be the most valuable single test when only a few fibers are available [14]. In 2019, Peets et al. stated that reflectance–FT-IR could be used to detect textile fibers using an FT-IR micro spectrometer in reflectance mode. The key advantages and limitations of these approaches were underlined, comparing the r–FT-IR and ATR–FT-IR spectra of textile fibers. However, ATR–FT-IR has been chosen to be the source of database inputs in this study, since it is fast, easy, and the most commonly used technique for textile fiber analysis [10,15,16,17,18,19,20,21,22]. In recent years, different chemometrics approaches have been used in conjunction with chemical data, such as principal component analysis (PCA), linear discriminant analysis (LDA), soft independent modelling by class analogy (SIMCA), cluster analysis, and correlation coefficient. Unscrambler is one of the commonly used software programs that is capable of performing multivariate statistical analysis on spectral data [23,24,25,26,27,28,29]. Spectroscopic data are handled by preprocessing methods and techniques such as Savitzky–Golay and Standard Normal Variate (SNV) [24,25,27,30,31,32,33,34,35]. In different fields other than forensics, Rismiwandira et al. used Unscrambler software to study the application of FT-IR spectroscopy for the detection of adulteration in palm sugar [27]. In 2019, Zhou et al. used the same software to analyze seven common types of fiber using the Near Infrared Spectroscopy method and the spectral data. They were able to develop PCA, PLS-DA, and SIMCA models that successfully determine a unique data pattern, facilitating the identification and classification of different types of fiber [28]. Undoubtedly, the use of different data science tools will improve the visual comparison of the collected fiber spectra, which will lead to enhancement of the evidential value of textile fibers. The purpose of this paper is to perform qualitative analysis on 138 man-made fibers (nylon, polyester, acrylic, and rayon) using ATR–FT-IR technique. The spectra of the analyzed fiber will be processed to develop data mining and classification models, including PCA and SIMCA, that aim to be promising forensic tools in fabrics discrimination. To our knowledge, the use of ATR–FT-IR techniques with chemometrics methods, including PCA and SIMCA, to analyze and classify four different types of synthetic fibers for forensic purposes distinguishes the work in this study for the first time.

## 2. Materials and Methods

### 2.1. Sampling and FT-IR Spectroscopy

A number of 138 synthetic fiber samples were supplied by microtrace manufacturers, which is an independent laboratory, headed by Skip Palenik, that specializes in microscopy and microchemistry for the identification of trace amounts of unknown compounds and single microscopic particles. For this study, the FT-IR Microscope “LUMOS–Bruker” was used to obtain the spectra of fiber samples. This instrument has a diamond crystal for attenuated total reflectance (ATR) data. Opus software (7.5) was used for collecting the spectrum in the mid infrared range of 4000–400 cm^−1^. Direct samples were taken and placed on ATR–FT-IR Crystal for the analysis; all samples were scanned to 100 scans with a resolution of 4 ${\mathrm{cm}}^{-1}$. To remove background effects on the samples, a background (air) measurement was performed; moreover, polystyrene film was used as a standard to ensure the performance of the instrument. After each sample analysis, to avoid cross contamination, the ATR crystal was cleaned with ethanol. Each sample was scanned for three trials and an average spectrum was obtained. After that, the spectra were smoothed automatically using OPUS software to enhance the FT-IR spectrum quality [36].

### 2.2. Data Analysis and Chemometrics

#### 2.2.1. Methodology–Software Package

**Figure 1:**
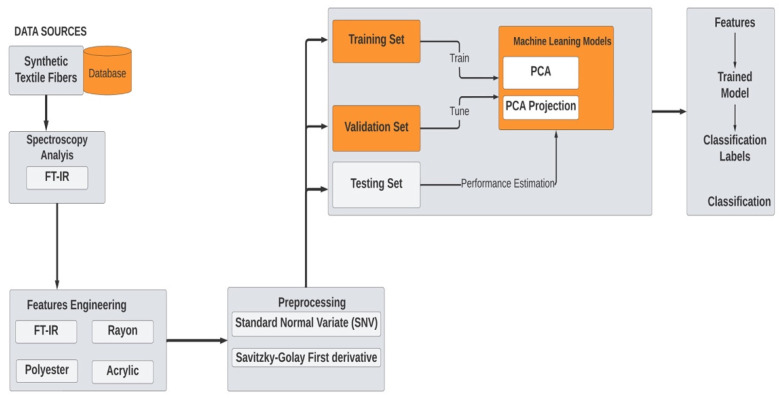
Data analysis methodology.

Multivariate data analysis was used to build classification models using spectral data by midinfrared spectroscopy. All data analysis steps were applied using Aspen Unscrambler software. The data analysis part involved several stages, including data selection, data cleaning, data characterization, data preprocessing, data mining, and data classification. In this study, the data analysis is composed by different steps, as presented in Figure 1.

#### 2.2.2. Data Selection

IR Spectra were obtained for 138 samples of FOUR different types of synthetic fibers: nylon, polyester, acrylic, and rayon. The data generated by the IR instrument are in the form OPUS. The Aspen Unscrambler software supports this form of data, so it was not necessary to convert the file to other forms such as CSV or EXL, etc. In addition, a regular IR spectrum for a single sample is displayed in a line plot, with the *y*-axis corresponding to transmittance in (%) and the *x*-axis corresponding to wavenumbers in (cm^−1^). The 138 samples were 48 nylon samples, 52 polyester samples, 26 acrylic samples, and 12 rayon samples. The data matrix consisted of 138 rows and 1753 columns.

#### 2.2.3. Data Cleaning

The next step was data cleaning. This step involved checking for duplicate data or missing columns and rows. No duplicate rows or columns were found. Additionally, no unassigned “NA” data were found.

#### 2.2.4. Data Characterization

The data were classified into four groups based on fiber type, based on previously known information about standard fibers. The four types were acrylic, nylon, polyester, and rayon. Therefore, an additional column was added to the data matrix and named “Type”. In addition, the data matrix was then divided into training and calibration sets. For each fiber type, approximately 70% of the data was selected for calibration (training) and 30% for testing. Thus, the training and test sets for each type were as follows:

- 26 acrylic datasets: 19 training, 7 tests;
- 12 rayon datasets: 9 training, 3 tests;
- 48 nylon datasets: 34 training, 14 tests;
- 52 polyester datasets: 37 training, 15 tests.

**Figure 2:**
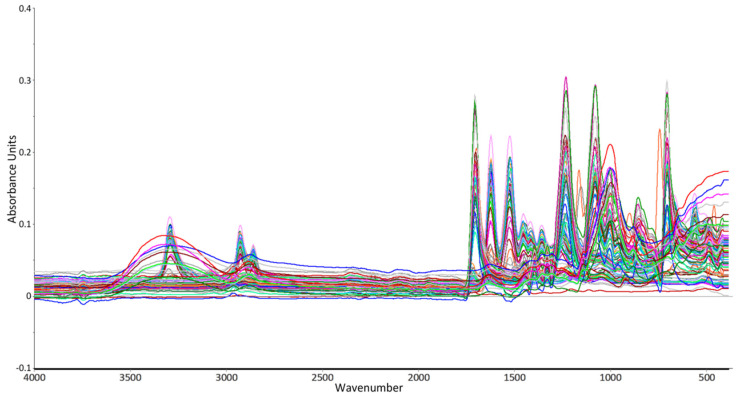
Spectra shape of all calibration samples.

A line plot was then created and shown in Figure 2 to observe the shape of the spectra for the calibration sets.

#### 2.2.5. Data Preprocessing

Raw data from the chemical procedures were subjected to preprocessing techniques using Savitzky–Golay and Standard Normal Variate (SNV) to transform the spectral data, minimize the effects of concentration on the characterization of the sample procedures, and reduce variability unrelated to the property of interest [23,24,25,27,28,35,37,38]. First, the data were transformed to their first derivative using Savitzky–Golay. 

In general, the Savitzky–Golay approach states that all successive subsets of 2 m + 1 are fitted by a polynomial of degree *p* (*p* ≤ 2 m), in the least squares sense [12]. The original data are differentiated in the middle by performing the differentiation d (0 ≤ d ≤ *p*) on the fitted polynomial instead of the original data. Finally, by combining the full input data with a digital filter of length 2 m + 1, the least squares fit of the polynomial can be performed quickly and easily.

**Figure 3:**
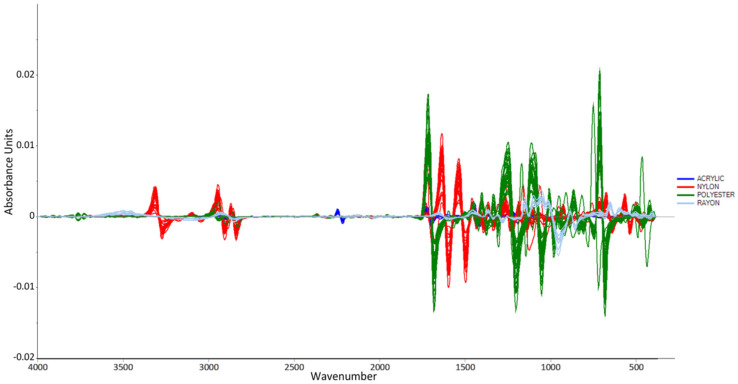
Calibration plot after performing Savitzky-Golay first derivative preprocessing technique.

**Figure 4:**
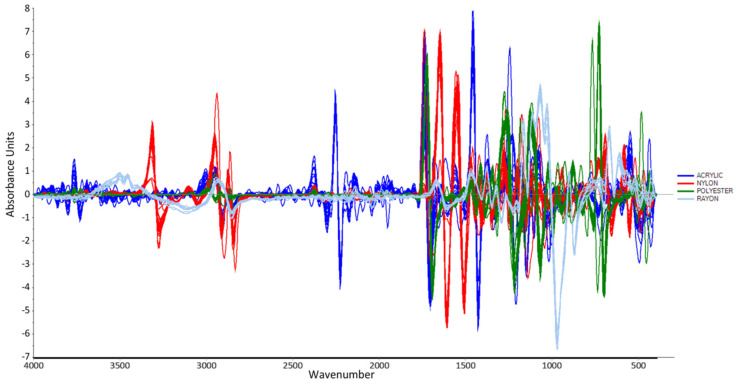
Calibration plot after performing Savitzky-Golay first derivative followed by SNV technique.

In this study, the number of smoothing points was set to 15 and the polynomial order was set to 2, since the spectra showed the best smoothing results when 15 smoothing points and 2 polynomial orders were applied. The derivative method was used in this work because it seemed to provide the best separation when performing PCA. In addition, the preprocessed data were subjected to another pretreatment, namely, the Standard Normal Variate (SNV) preprocessing technique. SNV calculates the average intensity value and subtracts it from each spectrum to normalize it [39]. The spectrum is then divided by the square root of the sum of squared intensities (the standard deviation). SNV was applied to improve the spectra and obtain the best results of separation when performing PCA by reducing the scattering effects. Figure 3 and Figure 4 show the line plots after performing the preprocessing techniques.

The preprocessing steps were essential before we moved on to the next step, which was the creation of the PCA model. When PCA is performed, the projection of the dataset is recalculated by PCA. The standard deviation of the variables is the basis for the new axis.

Consequently, a variable with a large standard deviation has more weight in the axis calculation than one with a small standard deviation. However, when the datasets are normalized, all variables have the same standard deviation, so they have the same weight and PCA calculates the appropriate axis.

## 3. Results and Discussion

### 3.1. Classification Models

#### 3.1.1. PCA

**Figure 5:**
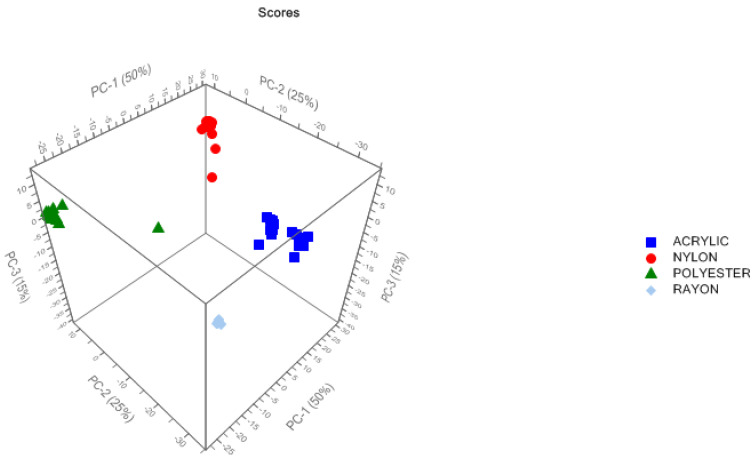
PCA model scores plot in 3D.

After performing the preprocessing steps, data were ready for modeling. First, calibration sets were subjected to PCA. The maximum number of components was set to be seven. However, it was indicated that only the first three components cover the majority of variance in the data, which means that only the first three components would have a significant contribution to the model. Figure 5 displays the distribution of the calibration samples along the three principal components.

**Figure 6:**
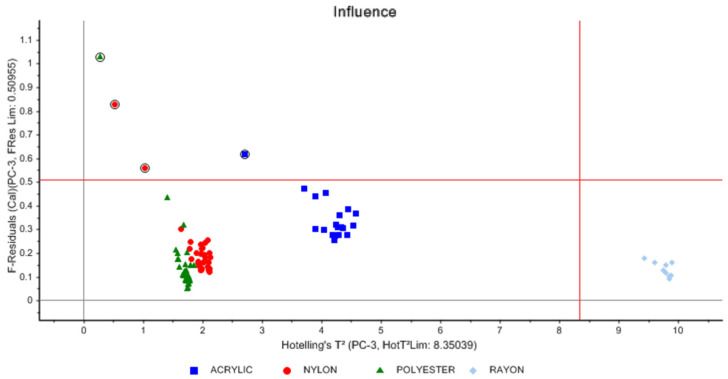
Influence plot.

The influence plot shows the Hotellings T^2^ values of the samples in the third principal component, as shown in Figure 6.

The influence plot helps identify possible outliers that could affect the model. Samples that lie above the horizontal line in the influence diagram are considered outliers that are not in the model, while samples that lie below the horizontal line and to the right of the vertical line are considered to be in the model, but extreme. In addition, any sample that lies above the horizontal line and to the right of the vertical line is considered an extreme outlier that would negatively affect the model. From the influence diagram in Figure 6, we were able to identify four outliers that were above the horizontal line and would greatly influence the model. Two of the outliers were nylon samples, one was polyester, and one was acrylic. These samples could also be observed to deviate from their groups in the scores plot. Therefore, the outliers were marked and then the PCA was recalculated without the marked samples. In addition, all rayon samples are distributed together to the right of the vertical line and below the horizontal line. This means that the rayon samples are extreme compared to other samples, which is very important information for the model to distinguish rayon from the other types.

#### 3.1.2. SIMCA and Cooman’s Plot

A PCA model was built separately for each type of the classified fiber samples; after that, Soft Independent Modeling of Class Analogies (SIMCA) was developed as a classification model for each test sample. The SIMCA model was able to classify all the samples correctly except for Nylon 117, Nylon 120, Polyester (167), and Polyester (174). Table 1 displays the results of SIMCA when applying a 5% significance limit.

**Figure 7:**
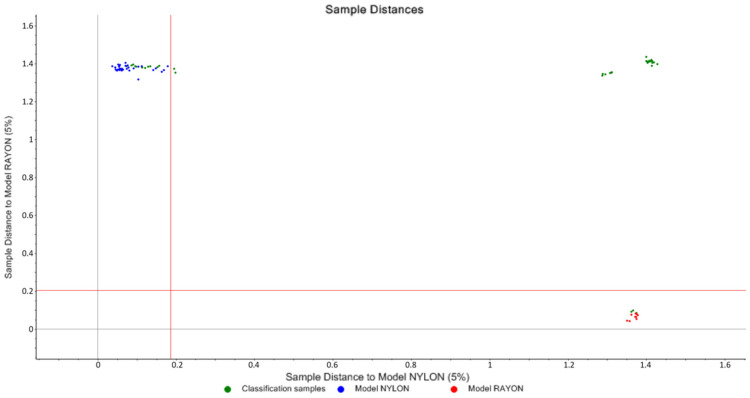
Nylon vs. rayon Cooman’s plot.

The findings of SIMCA are graphically displayed in a Cooman’s plot, which uses a pairwise comparison of the classes to examine the distance between the analyzed classes. To illustrate, Figure 7 displays a Cooman’s plot of nylon vs. rayon where the upper left quadrant belongs to the nylon group and the lower right quadrant belongs to the rayon group. No samples fell in the lower left quadrant, indicating that no sample was classified to both groups simultaneously. It was indicated that all the test samples were classified correctly. However, two nylon test samples were on the right of the vertical line, because the ordinate value for nylon classification is 0.190 when the significance limit of the distance to nylon model is set to 5%, and the ordinate values for the two samples were greater than 0.190. Thus, this explains why the two nylon samples were previously unclassified by the model in Table 1. However, the two samples could be classified as nylon because the sample distance is very close to the nylon model and very far from the other models.

**Figure 8:**
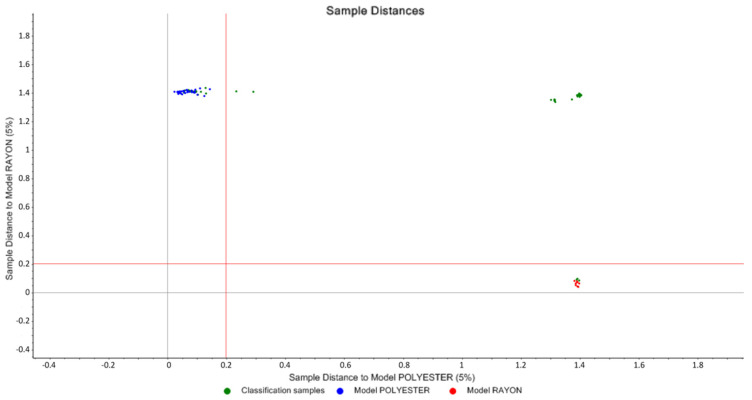
Polyester vs. rayon Cooman’s plot.

Figure 8 shows Cooman’s plot of polyester vs. rayon, with the polyester group in the upper left quadrant and the rayon group in the lower right quadrant. There were no samples in the lower left quadrant, signifying that no sample was categorized into both categories at the same time. All test samples were accurately categorized into their actual groups. However, two polyester test samples lie on the right of the vertical line, where the ordinate value for both samples is above 0.2, while the ordinate value of polyester classification is 0.20 when the significance limit of the distance to the nylon model is set to 5%. As a result, those two samples were not classified as polyester samples by the SIMCA method, as shown in Table 1. However, the given sample distance is extremely near to the polyester model and quite far from the other models, so both samples can be classed as polyester.

## 4. Conclusions

It has been found that fiber is the most dominant evidence that exists in crime scenes [9]. This study showed that the combination of spectroscopy and chemometrics has led to a highly desirable method for clustering and classifying 138 synthetic fiber samples into four groups. The Savitzky–Golay first derivative and SNV methods were used as preprocessing techniques for pretreatment of the data. All fiber samples in this work were clustered using a PCA model followed by SIMCA for classification purposes, where the model was able to classify 97.1% of the test samples successfully. Recently, comparing spectroscopic data by using different mathematical techniques has increased dramatically. The developed data mining models can become more extensive by adding more data of different fiber types, which will result in a sophisticated forensic tool for fiber discrimination. In order to achieve the best output of the established data analysis model, more data can be driven through the analysis of the same sample using different analytical techniques, such as Raman spectroscopy, micro spectrophotometry, and Mass spectrometry, coupled with chromatographic techniques.

## Figures and Tables

**Table 1:** SIMCA classification table.

Sample-Class	RAYON	NYLON	ACRYLIC	POLYESTER
SYN_NYLON_106		✓		
SYN_NYLON_117				
SYN_NYLON_118		✓		
SYN_NYLON_119		✓		
SYN_NYLON_120				
SYN_NYLON_17		✓		
SYN_NYLON_109		✓		
SYN_NYLON_110		✓		
SYN_NYLON_111		✓		
SYN_NYLON_112		✓		
SYN_NYLON_113		✓		
SYN_NYLON_114		✓		
SYN_NYLON_115		✓		
SYN_NYLON_116		✓		
SYN_POLYESTER_165				✓
SYN_POLYESTER_175				✓
SYN_POLYESTER_176				✓
SYN_POLYESTER_177				✓
SYN_POLYESTER_178				✓
SYN_POLYESTER_179				✓
SYN_POLYESTER_180				✓
SYN_POLYESTER_166				✓
SYN_POLYESTER_167				
SYN_POLYESTER_168				✓
SYN_POLYESTER_169				✓
SYN_POLYESTER_170				✓
SYN_POLYESTER_171				✓
SYN_POLYESTER_172				✓
SYN_POLYESTER_174				
SYN_RAYON_19	✓			
SYN_RAYON_19	✓			
SYN_RAYON_19	✓			
SYN_ACRYLIC_24			✓	
SYN_ACRYLIC_26			✓	
SYN_ACRYLIC_27			✓	
SYN_ACRYLIC_28			✓	
SYN_ACRYLIC_29			✓	
SYN_ACRYLIC_30			✓	
SYN_ACRYLIC_31			✓	

## Data Availability

Not applicable.

## Sample Availability

Samples of the compounds are not available from the authors.
